# 

**Published:** 1895-04

**Authors:** 


### *EDITORIAL.*

The office of THE JOURNAL is in room 330 Equitable Building.

The editors are not responsible for views expressed by contributors.

Articles for publication should reach this office not later than the 15th instant.

Address all communications, and make all remittances payable to THE ATLANTA MEDICAL AND SURGICAL JOURNAL, Atlanta, Ga.

Reprints of articles will be furnished, when desired, at a nominal cost. Requests for the same should always be made *on the manuscript.*

### THE MEDICAL ASSOCIATION.

The forty-sixth annual session of the Association will be held in Savannah, April 17th to 19th. Every one who can possibly attend should make it a point to do so. It is not necessary for us to remind our readers of the advantages which come from attending these annual meetings of the Association. Aside from that, the doctors of the State should endeavor, as far as they are individually and collectively able, to make the Association a great and grand success.

This meeting promises to be one of unusual interest, and socially speaking, the good people of Savannah will take care of their medical guests in a manner that will leave nothing to be desired.

Following is a partial list of papers to be read :

The Proper Care of School Children's Eyes. Dunbar Roy, Atlanta.

Backbone as a Mental Aseptic. J. S. Wimberly, Sanford.

Gonorrhea, its Effects and Treatment in the Female. J. W. Bailey, Gainesville.

One Hundred Consecutive Cases of Labor. H. McHatton, Macon.

A Remarkable Case of Ruptured Ectopic Gestation; Late Operation; Recovery. Geo. H. Noble, Atlanta.

Placenta Previa, Report of Case. M. L. Currie, Ailey.

A Rare Intestinal Tumor. L. G. Hardman, Harmony Grove.

Reports of Several Interesting Operations. W. S. Armstrong, Atlanta.

Graves' Disease, with Cases. J. M. Hull, Augusta.

Ligation of External Carotid Artery as Preliminary to and Coincident with Operations upon the Jaws, and Report of Case. Wm. Perrin Nicolson, Atlanta.

The Limits of Conservatism in Pelvic Pus-Cases. Ross P. Cox, Rome.

Mind and Body. Charles S. Webb, Atlanta.

A Ready Method of Resuscitation in Suspended Animation. P. L. Hillsman, Albany.

A Case of Cancer of Pancreas, with Autopsy. Valentine H. Taliaferro, Atlanta.

Mental Suggestion as an Aid in the Treatment of Morphinomania—Cases in Evidence. Samuel H. Green, Oakdale.

Wounds from Explosives—Their Treatment. T. D. McKown, Chickamauga.

Obstetrics as Practiced in the Mountains of North Georgia. T. M. Greenwood, Mineral Bluff.

Some Observations with Regard to the Treatment of Typhoid Fever. A. C. Davidson, Sharon.

The Cause and Treatment of Corneal Ulcers. S. Latimer Phillips, Savannah.

Traumatic Wounds of the Eye and their Treatment. T. E. Mitchel, Columbus.

The Topical Treatment of Chronic Circumscribed Eczema. Bernard Wolff, Atlanta.

Galvanism in Office Practice. J. C. LeHardy, Savannah.

Obstetrics in Vienna, Austria. Wm. B. Gilmer, Macon.

Urinalysis. Louis H. Jones, Atlanta.

Title to be given. F. M. Ridley, LaGrange.

The Surprises We Meet in Abdominal Surgery. Seth N. Jordan, Columbus.

Something New in the Treatment of Women after Confinement. R. J. Nunn, Savannah.

Title to be announced. C. D. Hurt, Atlanta.

Report of My Surgical Work for Past Six Months, with Some Deductions. J. B. S. Holmes, Atlanta.

History of Medicine and Surgery in Georgia. L. B. Grandy, Atlanta.

Officers are: W. F. Westmoreland, Atlanta, Ga., President; R. H. Taylor, Griffin, Ga., Vice-President; W. Tate, Tate, Ga., Vice-President; E. C. Goodrich, Augusta, Ga., Treasurer; D. H. Howell, Atlanta, Ga., Secretary.

